# Spinal astrocytes involved in the pathogenesis and treatment of neuropathic pain

**DOI:** 10.3389/fncel.2025.1547524

**Published:** 2025-02-21

**Authors:** Xiangmiao Li, Yushan Huang, Jinzhu Bai

**Affiliations:** ^1^School of Rehabilitation, Capital Medical University, Beijing, China; ^2^Department of Spine and Spinal Cord Surgery, Beijing Boai Hospital, China Rehabilitation Research Center, Beijing, China; ^3^School of Orthopedics, Capital Medical University, Beijing, China

**Keywords:** astrocytes, neuropathic pain, pathogenesis, treatment, spinal cord

## Abstract

Neuropathic pain is a common and severe type of chronic pain, and its pathogenesis has not been fully defined. Increasing evidence shows that spinal astrocytes play indispensable roles in the occurrence and development of neuropathic pain. Most studies have suggested that activated astrocytes can crosstalk with other glial cells and neurons through morphological and functional changes, exacerbating the development of pain. However, reactive astrocytes have a dual role. As a defense mechanism, reactive astrocytes have roles in increasing neuroprotection and stimulating neurogenesis. Studies have demonstrated a potentially beneficial role for astrocyte activation in neuropathic pain. In addition, the therapeutic mechanisms of multiple drugs and neuromodulatory techniques are thought to be related to astrocytes. This review highlights the recent advances and significance of spinal astrocytes, emphasizing the need for a better understanding of their roles in the pathogenesis and treatment of neuropathic pain.

## 1 Introduction

Neuropathic pain (NP) refers to pain that arises as a direct consequence of a lesion or disease affecting the somatosensory system and is a common chronic severe pain (Burke et al., 2017). The initial affected site can be categorized into central NP (chronic central poststroke pain, chronic central NP associated with spinal cord injury, chronic central NP associated with multiple sclerosis, etc.) and peripheral NP (trigeminal neuralgia (TN), chronic NP after peripheral nerve injury, postherpetic neuralgia, etc.) (Szok et al., 2019). Its signs and symptoms include allodynia, hyperalgesia, and paresthesia (Cavalli et al., 2019), which seriously affect the quality of life of patients and increase the prevalence of depression, anxiety, and other adverse emotions (Khan et al., 2020). Currently, the pathogenesis of NP has not been fully clarified, and the processing and regulation of pain signals are coordinated by complex structures, specific pathways, and different types of cells (Chen Y. L. et al., 2022).

Astrocytes account for approximately 30% of the cells in the mammalian central nervous system (CNS) and perform many vital functions (Lee et al., 2022). Under physiological conditions, astrocytes monitor the balance of the synaptic cleft and provide essential structural and metabolic support to neurons. After noxious stimulation and nerve injury, the phenotype, function and gene expression of astrocytes will change significantly, which is called reactive astrogliosis (Li et al., 2019). During this process, naive astrocytes undergo typical changes such as process extension, hypertrophy, and increased GFAP expression, thus forming the phenotypic characteristics of reactive astrocytes (Li et al., 2019). After that, reactive astrocytes proliferate, migrate and transform into scar-forming astrocytes (Li et al., 2019). Reactive astrogliosis has a dual role. As an initial defense mechanism, reactive astrocytes can increase neuroprotection and nutritional support to insult-stressed neurons, and can prevent the spread of lesions by forming a physical barrier between lesions and neighboring tissues (Pekny et al., 2014). However, reactive astrocytes also have deleterious functions. After activation, astrocytes have functional changes, unable to play normal homeostatic functions such as neural support and neurotransmitter uptake, energy metabolism, ion balance and immune regulation, and promote pathological progress by releasing toxic factors such as inflammatory cytokines and neurotoxic lipids (Patani et al., 2023).

Studies on the mechanisms of astrocyte involvement in NP have received increasing attention. Some research has focused on spinal astrocytes, especially reactive astrocytes in the spinal dorsal horn (SDH), which play key roles in the development and maintenance of pain. When conditions such as central nervous system hypoxia, peripheral nerve injury and neurodegeneration occur, they can induce the transformation of spinal astrocytes into reactive astrocytes, which triggers harmful morphological and functional alterations, driving the development of NP (Mika et al., 2013; Ji et al., 2019). Astrocyte activation and its induced neuroinflammation are key factors in the generation and persistence of NP (Li J. et al., 2022). Pathways or factors such as JAK/STAT, NF-κB, PI3K/AKT, Nrf2, TGF-*β*1, Wnt/β-catenin, C/EBPβ, etc. in astrocytes are involved in the regulation of astrocyte to reactive astrocytes. The changes in the expression of a variety of receptors and proteins in reactive astrocytes promote the development of NP, such as the decrease in the expression of glutamate transporters, leading to excessive glutamate transmission and neuroadaptive changes, which aggravate pain (Nakagawa and Kaneko, 2010; Benarroch, 2022). Reactive astrocytes also regulate CNS inflammation by secreting proinflammatory or anti-inflammatory cytokines, chemokines and proteases. In addition, these mediators are also involved in the interaction between astrocytes and microglia and neurons, altering neural excitability by modulating excitatory and inhibitory synaptic transmission, leading to central sensitization and NP (Ji et al., 2019).

Given the important role that astrocyte activation plays in the maintenance and progression of NP, targeting astrocytes for treatment is a worthy line of investigation. In recent years, research evidence has identified inhibition of inflammatory mediator release or cellular downstream chemokine and cytokine signaling through signaling pathways, hemichannels, or purinergic receptors within astrocytes as the analgesic target of several commonly used drugs in the clinic. It has also been found that targeting astrocyte signaling pathways is also an important way to improve the efficacy of analgesic drugs and reduce side effects. A variety of other novel drugs targeting astrocyte activation are being studied and developed. In addition to pharmacological treatments, a number of non-pharmacological mechanisms are currently being investigated in animal models, including exercise and neuromodulation (including spinal cord stimulation and photobiomodulation), and may offer ways to control chronic pain through modulation of astrocyte responses and neuroinflammation.

## 2 Involvement of spinal astrocytes in NP development

### 2.1 Astrocyte activation promotes the development and occurrence of NP

Astrocytes are central players leading to chronic pain states and have extensive contacts with interneuronal synapses (Ji et al., 2019). When stimulated by external factors (nerve injury, viral infections, etc.) or by the release of substances (nitric oxide, prostaglandins, etc.) from secondary neurons that transmit pain, astrocyte activation results in alterations in cell morphology, homeostatic metabolism, and gene expression profiles, referred to as “reactive astrocytes” (Chen Y. L. et al., 2022). Garrison et al. (1991) first observed that astrocytes on the injured side of the spinal cord in rats changed from a resting phenotype to an active phenotype after sciatic nerve injury and systemically released cytokines such as tumor necrosis factor-*α* (TNFα) and interleukin-1β (IL-1β) at the spinal cord level.

Since then, an increasing number of studies have shown that activated astrocytes can establish glial–neuronal and glial–glial connections with neurons and microglia by secreting and releasing a variety of cytokines, activating intracellular protein kinases, altering the expression of receptors or channel proteins, and inducing other functional alterations, which together mediate the development and maintenance of NP. Astrocytes may also play a crucial role in chronic pain and CNS disease through morphological changes such as cell swelling (Zhou et al., 2019; He et al., 2024).

#### 2.1.1 Multiple signaling pathways are involved in the development of NP

Multiple signal transduction pathways, such as the mammalian target of rapamycin (mTOR) pathways, signal transducer and activator of transcription 3 (STAT3) pathways, nuclear factor kappa-light-chain-enhancer of activated B cells (NF-κB) pathways, and mitogen-activated protein kinase (MAPK) pathways, are involved in the process of transforming resting astrocytes into reactive astrocytes. Activated astrocytes can induce neuronal hypersensitivity by releasing a variety of inflammatory factors (cytokines, chemokines, proteases, etc.), thereby mediating NP. In recent years, scholars have conducted more in-depth research and explorations of the upstream and downstream molecules of these pathways, which further supplements the previous research results (Table 1).

**Table 1:** Astrocyte signaling pathways in neuropathic pain.

Name	Upstream	Downstream	Pain model	Species	Mechanisms	Refs
mTOR	TRPM7	-	SCI	rat	Promote the activation of astrocytes. Induce and/or maintain NP.	Kim et al. (2024)
JAK2/STAT3	IL-6	-	SCI	rat	Induce the activation of astrocytes. Promote the occurrence of mechanical allodynia and thermal hyperalgesia.	Lee et al. (2023)
		-	CCI-ION	rat	CSF IL-6 diffuses original NP to widespread pain. Activate astrocyte STAT3 signaling.	Yu et al. (2024)
	ZDHHC23/CXCL-10、ZDHHC23/IL-6、ZDHHC23/GM-CSF	-	NCP	rat	Promote the activation and proliferation of astrocytes. Participate in the maintenance of pain.	Fan et al. (2023)
STAT3	BMP4	-	PHN	rat	Promote astrocyte activation through STAT3 signaling to induce allodynia.	Chen et al. (2024)
ERK and p38	FoxO1-AQP5 axis	-	CCI	rat	Promote the activation of spinal astrocytes and microglia. Increase the production of inflammatory mediators.	Yu et al. (2022)
JNK	TNF α	HDAC2、GLT-1	SNL	rat	Enhance synaptic glutamatergic neurotransmission. Involve in the maintenance of NP.	Lu et al. (2021)
	RIP3	-	CCI	rat	Promote neuronal damage by upregulating JNK. Promote inflammatory responses.	He et al. (2021)
Wnt10a/β-catenin			CCI	rat	Promote astrocyte activation and the subsequent release of inflammatory mediators.	Zhao et al. (2021)
miR-130a-5p/CXCL12/CXCR4	lncRNA MEG3	Rac1、NF-κB pathway	SNL、 CCI	Mouse、rat	Exacerbate NP and intensify neuroinflammation.	Dong et al. (2021a) and Dong et al. (2021b)
BDNF/TrkB			PCI	mouse	Promote the activation of astrocytes.	Phan et al. (2023)

TRPM7, transient receptor potential melastatin 7; SCI, spinal cord injury; NP, neuropathic pain; IL-6, interleukin-6; CCI-ION, chronic constriction injury of the infraorbital nerve; CSF, cerebrospinal fluid; ZDHHC23, palmitoyltransferase ZDHHC23; CXCL-, C-X-C motif chemokine ligand-; GM-CSF, granulocyte–macrophage colony-stimulating factor; NCP, neuropathic cancer pain; BMP4, bone morphogenetic protein 4; PHN, postherpetic neuralgia; FoxO1, forkhead box O1; AQP5, aquaporin 5; HDAC2, histone deacetylase 2; GLT-1, glutamate transporter-1; SNL, spinal nerve ligation; CXCR4, C-X-C motif chemokine receptor 4; BDNF, brain-derived neurotrophic factor; TrkB, tropomyosin receptor kinase B; PCI, partial crush injury.

Spinal cord injury (SCI) can upregulate the expression of transient receptor potential melastatin 7 (TRPM7) in SDH astrocytes and promote the activation of astrocytes by activating the mTOR signaling pathway, thereby inducing and/or maintaining NP (Kim et al., 2024). The application of TRPM7 inhibitors or mTOR signaling pathway inhibitors can significantly inhibit astrocyte activation and reduce the production of pain-related factors, thereby alleviating NP (Kim et al., 2024).

At the level of the spinal cord, the Janus kinase (JAK)/STAT3 pathway plays an important role in the activation of glial cells. In the early stage, Kohro et al. (2015) confirmed that JAK-STAT3 signaling pathway of SDH astrocytes was involved in the maintenance of NP by applying minimally-invasive SDH microinjection technique. Later studies found that interleukin-6 (IL-6) is the ligand activating this pathway. After SCI, IL-6 can mediate the activation of the JAK2/STAT3 signaling pathway in spinal astrocytes and microglia and promote the occurrence of mechanical allodynia and thermal hyperalgesia by inducing the activation of astrocytes and microglia (Lee et al., 2023). Fan et al. (2023) reported that SDH astrocytes are activated with disease progression and the onset of cancer pain in a neuropathic cancer pain (NCP) model. Upregulation of the palmitoyltransferase ZDHHC23 leads to increased levels of palmitoylation of glial fibrillary acidic protein (GFAP) and increased secretion of inflammatory factors such as C-X-C motif chemokine ligand 10 (CXCL-10), IL-6, and granulocyte–macrophage colony-stimulating factor (GM-CSF) (Fan et al., 2023). These factors further promote the activation and proliferation of astrocytes by activating the STAT3 signaling pathway, thus participating in the maintenance of pain. Yu et al. (2024) reported that chronic constriction injury of the infraorbital nerve (CCI-ION) can lead to an increase in IL-6 levels in the cerebrospinal fluid and that IL-6 can spread from primary NP to generalized pain of the distal spinal cord segment by activating STAT3 signal transduction in the SDH astrocytes of the distal lumbar segment. Chen et al. (2024) confirmed that in postherpetic neuralgia (PHN), cerebrospinal fluid-derived bone morphogenetic protein 4 (BMP4) may also promote astrocyte activation through STAT3 signaling to induce allodynia.

MAPKs are a class of evolutionarily conserved intracellular signaling molecules. This family consists of three main members: extracellular regulated protein kinase (ERK), p38 and JNK. Existing evidence suggests that the MAPK family plays an important role in regulating neuroplasticity and the inflammatory response (Ji et al., 2009). Nerve injury or SCI induces MAPK activation in spinal cord glial cells, which subsequently plays an important role in the development and maintenance of NP (Ji et al., 2009). The expression of p-ERK in glial cells is highly dynamic after nerve injury, with a sequence of transient ERK activation in neurons inducing ERK activation in early microglia and then inducing ERK activation in late astrocytes through glial–glial interactions (Zhuang et al., 2005; Ji et al., 2009). Ma et al. (2022) found that p-ERK was expressed mainly in SDH astrocytes on the 29th day after spared nerve injury (SNI) and that SNI induced significant activation of ERK in astrocytes but not microglia, which further verified the role of astrocytes in the maintenance of pathological pain. Yu et al. (2022) confirmed that the forkhead box O1 (FoxO1)/aquaporin 5 (AQP5) pathway in astrocytes and microglia can activate the ERK and p38 MAPK pathways, promote the activation of spinal astrocytes and microglia, and increase the production of inflammatory mediators in spinal cord tissue, thus inducing NP. Aberrant activation of the JNK signaling pathway in astrocytes plays an important role in the maintenance of NP. After SNL, JNK in spinal astrocytes is slowly and continuously activated and phosphorylated to the JNK1 isoform, which leads to the persistence of mechanical allodynia (Zhuang et al., 2006). Recently, new studies have shown that upregulation of histone deacetylase 2 (HDAC2) expression and decreased glutamate transporter-1 (GLT-1) expression in spinal astrocytes are downstream events of JNK activation, and that the above events are involved in the maintenance of NP by enhancing synaptic glutamatergic neurotransmission in the SDH (Lu et al., 2021). He et al. (2021) found that receptor interacting serine/threonine kinase 3 (RIP3) induces JNK signaling transmission, thus RIP3 may be a new therapeutic target for NP patients.

Sciatic nerve injury upregulates maternally expressed gene 3 (MEG3) expression in dorsal spinal astrocytes. MEG3 can upregulate the expression of components of the C-X-C motif chemokine ligand 12 (CXCL12)/ CXC motif chemokine receptor 4 (CXCR4)/ ras-related C3 botulinum toxin substrate 1 (Rac1) signaling axis and activate the toll-like receptor 4 (TLR4)/NF-κB pathway by inhibiting the expression of miR-130a-5p, thus promoting the activation of astrocytes and aggravating NP (Dong et al., 2021a). The upregulation of miR-130a-5p expression can regulate CXCL12/CXCR4-mediated astrocyte activation and inactivate the downstream Rac1, NF-κB and ERK signaling pathways to reduce the development of NP (Dong et al., 2021b). Other studies have confirmed that the brain-derived neurotrophic factor (BDNF)/ tropomyosin receptor kinase B (TrkB) signaling pathway (Phan et al., 2023) and the Wnt Family Member 10A (Wnt10a)/*β*-catenin signaling pathway (Zhao et al., 2021) can also promote the occurrence of NP by mediating the activation of spinal astrocytes and the subsequent release of inflammatory mediators.

#### 2.1.2 Receptors, ion channels and transporters involved in the development of NP

A variety of receptors, ion channels, transporters, and intracellular kinases in astrocytes play important roles in the regulation of pain after injury through changes in their expression.

Aquaporin 4 (AQP4) is an ion channel that is expressed mainly in the endfeet of astrocytes and is involved in metabolic waste excretion. Several studies have demonstrated that the upregulation of AQP4 expression in SDH astrocytes can promote their activation, thus contributing to the development and maintenance of the NP (Guo et al., 2022; Song et al., 2024). He et al. (2024) reported that AQP4 is also involved in the morphological changes that occur after astrocyte activation. Aurora kinase B (AURKB) regulates the phosphorylation of nuclear factor of activated T cells 5 (NFAT5) and promotes its translocation into the nucleus, thereby stimulating AQP4 expression after peripheral nerve injury (He et al., 2024). The above process leads to astrocyte swelling and promotes the development of NP.

**Figure 1:**
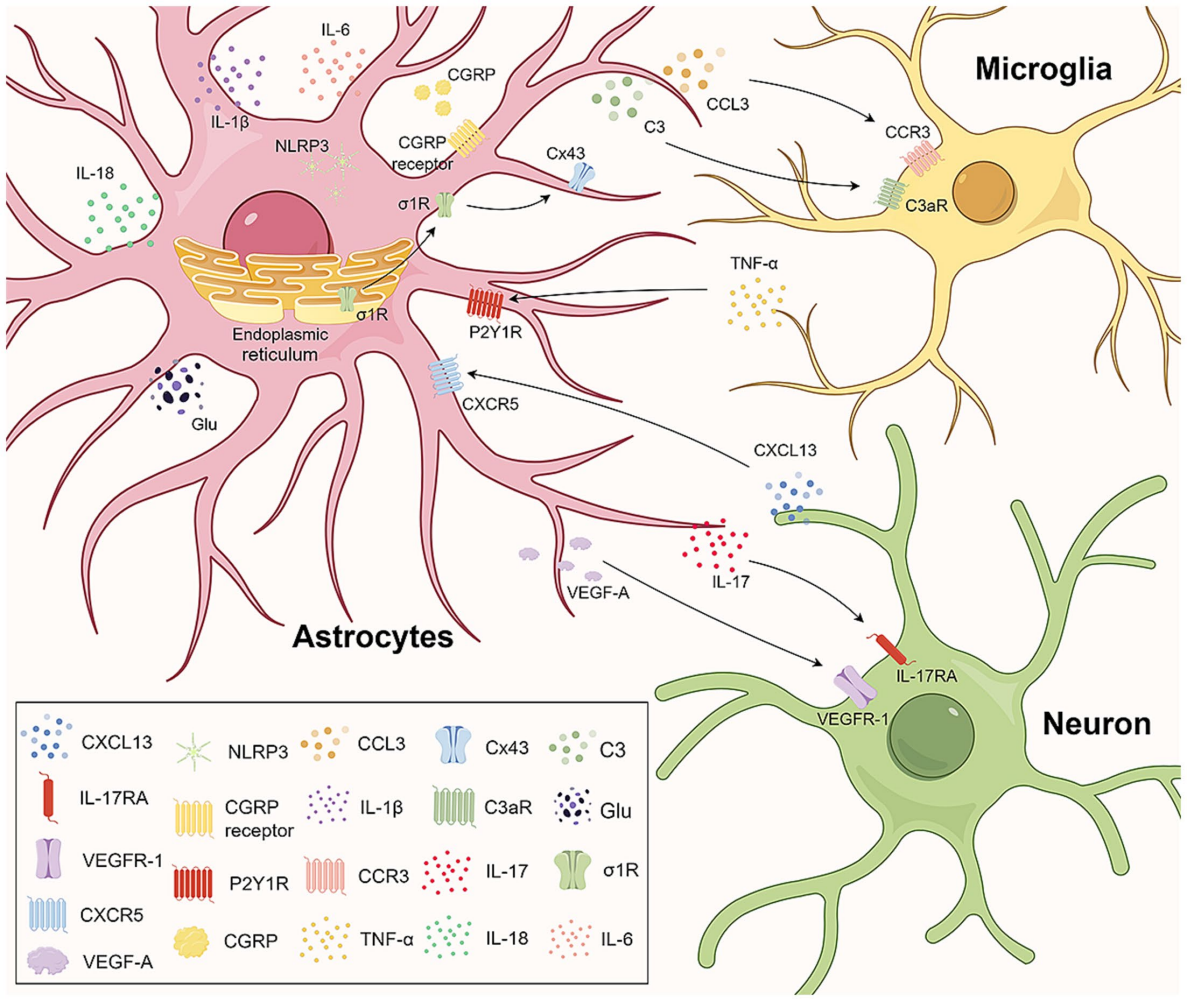
Astrocytes engage in crosstalk with microglia and neurons. In the central nervous system, astrocytes engage in crosstalk with microglia and neurons through ligand–receptor interactions and jointly mediate the occurrence of neuropathic pain. CGRP upregulation in the spinal cord induces the upregulation of IL-1β, IL-6, CCL3, NLRP3 in astrocytes and CCR3 in microglia by binding to the CGRP receptor. CCR3-CCL3 signaling aids astrocyte-microglia communication. The C3 in astrocytes and C3aR in microglia increase, facilitating crosstalk via C3/C3aR. The activated σ1R on astrocytes transfers to the plasma membrane, interacting with Cx43 for astrocyte-microglia communication. Microglia-released TNF-α upregulates P2Y1Rs in astrocytes, promoting glutamate release. Neuronal CXCL13 activates astrocytes, targeting NLRP3 to upregulate IL-1β, IL-18, and neuropathic pain. IL-17 from astrocytes regulates neuroplasticity and neuropathic pain via IL-17RA neurons. VEGF-A in astrocytes stimulates nociceptive neurons via VEGFR-1, involved in neuropathic pain pathophysiology. CGRP, calcitonin gene-related peptide; IL-1β, interleukin-1β; IL-6, interleukin-6; CCL3, CC-chemokine ligand 3; NLRP3, NOD-like receptor 3; CCR3, C-C motif chemokine receptor 3; C3aR, complement C3a receptor; σ1R, sigma-1 receptor; ER, endoplasmic reticulum; Cx43, connexin 43; TNF-α, tumor necrosis factor α; P2Y1R, human purinergic G protein-coupled receptor P2Y1; CXCL13, chemokine C-X-C motif ligand 13; CXCR5, C-X-C chemokine receptor type 5; IL-17, interleukin-17; IL-18, interleukin-18; IL-17RA, interleukin 17 Receptor A; VEGF-A, vascular endothelial growth factor A; VEGFR-1, vascular endothelial growth factor receptor-1.

P2 purinergic receptors are widely distributed in a variety of tissues and systems throughout the body and are classified into P2X and P2Y receptors according to their molecular structures and signaling mechanisms. Several studies have shown that astrocyte P2 receptors are involved in the development and maintenance of NP. Song et al. (2023) reported that in an NP model established by chronic compression of the dorsal root ganglion (CCD), the upregulation of astrocyte P2Y2 receptor protein expression can increase the expression of transient receptor potential vanilloid 4 (TRPV4), which leads to an increase in the intracellular Ca^2+^ concentration in astrocytes and the activation of astrocytes, thus mediating the occurrence of NP. Liu S. et al. (2023) found that spinal nerve ligation (SNL) induces the release of TNF-*α* by microglia, which promotes the upregulation of P2Y1R expression in SDH astrocytes. This process leads to an increase in A1-like polarization of astrocytes and affects Ca^2+^ signaling, which subsequently facilitates glutamate release from astrocytes and ultimately leads to NP (Figure 1).

The expression of Connexin 43 (Cx43), a major connexin protein expressed by astrocytes, has been reported to be significantly increased during NP. Previous studies have suggested that astrocyte Cx43 mediates NP by releasing chemokines after nerve injury (Chen et al., 2014). Recently, Luo et al. (2023) reported that Cx43 deficiency can inhibit spinal c-Fos expression while significantly reducing the degree of spinal microglial activation induced by nerve injury. These findings suggest that astrocyte Cx43 may affect the severity of NP by regulating microglial activation, but the specific mechanism needs to be further investigated. Zhu et al. (2022) showed that streptozotocin (STZ)-induced type I diabetes results in the sustained upregulation of chemokine CXC receptor 4 (CXCR4) and Cx43 in both astrocytes and neurons. The continuous increase in CXCR4 expression might induce neuronal excitability, whereas Cx43 might mediate intercellular inflammatory signaling so that dysfunctional astrocytes cannot counteract inflammatory factors during the late phase of diabetes, which leads to the occurrence and aggravation of diabetic neuropathic pain (DNP) (Zhu et al., 2022).

Sphingolipids are an important class of biologically active signaling molecules that are abundantly expressed in the CNS, and their metabolites, such as sphingomyelin and sphingosine-1-phosphate (S1P), have important roles in NP. Metabolomics has shown that dysregulation of sphingolipid metabolism in the SDH plays an important role in the maintenance of NP (Patti et al., 2012). Several studies have shown that dysregulation of S1P expression drives NP through activation of S1P receptor 1 (S1PR1) in SDH astrocytes. In animal models of NP induced by bortezomib and peripheral nerve injury, increased levels of S1P expression in SDH promoted NP by activating astrocyte S1PR1, leading to an increase in glutamate release from presynaptic terminals and release of the pro-inflammatory cytokine IL-1β (Stockstill et al., 2018; Chen et al., 2019). Liu B. et al. (2023) found that aminoacylase 1 (ACY1) expression was increased in SDH neurons in mouse models of SNI-induced NP. ACY1 interacted with sphingosine kinase 1 (SphK1) and facilitated its transportation to the plasma membrane, leading to the production of S1P on the cell membrane. Up-regulated S1P selectively activated astrocytes via S1PR1, which promoted the release of cytokines and chemokines and promoted NP.

SNI can decrease bile acid levels while increasing the expression of the bile acid receptors takeda G protein-coupled receptor 5 (TGR5) and nuclear farnesoid X receptor (FXR) in SDH glial cells and GABAergic neurons (Wu et al., 2023). The activation of TGR5 or FXR inhibits glial cell activation and p-ERK expression in the SDH, which in turn enhances the extrasynaptic inhibitory effect of GABAA receptors, thereby alleviating mechanical allodynia. Dipeptidyl-Peptidase 4 (DPP4) is a membrane glycoprotein expressed in many cell types. DPP4 can interact with Toll-like receptor 4 (TLR4) signaling in SDH astrocytes and increase the expression of IL-6 and TNF-*α*, thereby promoting inflammatory pain (Kozsurek et al., 2023).

Several other studies have shown that the expression of various receptors and ion channels, such as the sigma-1 receptor (Sig-1R) (Choi et al., 2021; Choi et al., 2022), ion channel LRRC8A (Ding et al., 2022), transient receptor potential vanilloid 1 (TRPV1) (Baba et al., 2021; Dalenogare et al., 2023), sulfonylurea receptor 1 (SUR1) (Tsymbalyuk et al., 2021), and calcitonin gene-related peptide (CGRP) receptor (Sun et al., 2021), is upregulated in astrocytes in the NP model (Table 2). These overexpressed receptors and channels promote the development and maintenance of NP by mediating the activation of spinal astrocytes and neurons, regulating calcium signaling, activating protein kinases, releasing inflammation-related signaling molecules, participating in astrocyte proliferation and autophagy, and affecting the expression of inflammation-related genes. The expression of some receptors and ion channels is also inhibited. In a model of NP induced by oxaliplatin (OXA), the suppression of adenosine receptor A1 (A1R) expression in spinal astrocytes leads to impaired glutamate clearance and synaptic glutamate accumulation, which induces mechanical and cold allodynia in animals treated with OXA (Liu L. et al., 2023). Reduced expression of methylated CpG-binding protein 2 (MeCP2) following chronic constriction injury (CCI) leads to decreased expression of inwardly rectifying potassium channel 4.1 (Kir4.1) in spinal astrocytes (Ou et al., 2023). This change results in an increase in the extracellular K level that enhances the sustained tonic firing of pain relay neurons, leading to hyperalgesia in the chronic phase of NP.

**Table 2:** Astrocyte-associated ion channels and receptors in neuropathic pain.

Name	Function	Pain model	Species	Expression changes	Mechanisms	Refs
Receptors						
Sig-1R	Chaperone receptor	CCI	mouse	↑	Regulates intracellular Ca^2+^ responses. Participates in NMDA receptor GluN1 phosphorylation. Increases mechanical allodynia.	Choi et al. (2021), Choi et al. (2022)
TRPV1	Capsaicin receptor	pSNL RR-EAE PMS-EAE	mouse	↑	Contributes to the maintenance of astrogliosis. Maintains heat hypersensitivity. Increases mechanical and cold hypersensitivity.	Baba et al. (2021), Dalenogare et al. (2023)
SUR1	Form K_ATP_ channels	Sciatic nerve cuffing	mouse	↑	Co-assembles with TRPM4 to form SUR1-TRPM4 channels. Participates in mechanical allodynia and thermal hyperalgesia.	Tsymbalyuk et al. (2021)
CGRP receptor	G-protein-coupled receptors	CCI	rat	↑	Binds to CGRP and leads to H3K9ac. Promotes neuropathic pain.	Sun et al. (2021)
Ion channels						
LRRC8A channel	Anion channels	EAE	mouse	↑	Mediates the reactivity of astrocytes by regulating the STAT3 pathway. Participates into the pain hypersensitivity.	Ding et al. (2022)

↑, upregulated. Sig-1R, sigma-1 receptor; CCI, chronic constriction injury; TRPV1, transient receptor potential vanilloid 1; pSNL, partial sciatic nerve ligation; RR-EAE, relapsing–remitting experimental autoimmune encephalomyelitis; PMS-EAE, progressive experimental autoimmune encephalomyelitis; SUR1, sulfonylurea receptor 1; TRPM4, transient receptor potential melastatin 4; CGRP, calcitonin gene-related peptide; H3K9ac, histone H3 lysine 9 acetylation; EAE, experimental autoimmune encephalomyelitis.

In the CNS, astrocytes can also engage in crosstalk with other cells, such as microglia and neurons, through ligand–receptor associations, which together mediate NP (Figure 1). Neuronal production of C-X-C motif chemokine 13 (CXCL13) promotes NP by binding to the astrocyte receptor C-X-C Motif Chemokine Receptor 5 (CXCR5) and activating astrocytes (Jiang et al., 2016). Based on this finding, Yi et al. (2024) observed that the CXCL13/CXCR5 signaling pathway can target the activation of the NOD-like receptor protein 3 (NLRP3) inflammasome in spinal astrocytes, leading to the upregulation of the inflammatory factors IL-1β and interleukin-18 (IL-18), which promote NP in rats with chronic postsurgical pain (CPSP). Cisplatin administration can induce the expression of calcitonin gene-related peptide (CGRP) in the spinal cord (Xie et al., 2024). Increased CGRP expression induces astrocyte activation and the upregulation of IL-1β, IL-6, CC-chemokine ligand 3 (CCL3) and NLRP3 expression by binding to CGRP receptors on spinal astrocytes and upregulating C-C motif chemokine receptor 3 (CCR3) expression in microglia. CCR3-CCL3 signaling may be involved in the communication between astrocytes and microglia and promote the occurrence and development of chemotherapy-induced neuropathic pain (CIPN). Inhibition of sigma-1 receptor (σ1R) can attenuate CCI-induced proliferation and apoptosis signaling pathways in SDH astrocytes and reduce the release of inflammatory cytokines (Denaro et al., 2023). It is also able to downregulate Cx43 expression levels, which significantly reduces microglia–astrocyte communication and attenuates NP.

#### 2.1.3 Cell-derived mediators involved in the development of NP

Astrocyte activation can regulate the immune microenvironment of the CNS by releasing various signaling molecules, such as ATP (Ishikura et al., 2021), cytokines, chemokines, growth factors, proteases (Kawasaki et al., 2008) and other mediators, which mediate the development of NP (Table 3).

**Table 3:** Astrocyte-derived mediators in neuropathic pain.

Name	Function	Pain model	Species	Expression changes	Mechanisms	Refs
Cytokines						
IL-1β	Interleukin-1	Bone cancer and CCI	rat	↑	Pro-IL-1β cleaves into the active form of IL-1β. Promote the development and maintenance of persistent hyperalgesia.	Zhang et al. (2005), Green-Fulgham et al. (2024)
IL-17	Interleukin-1	SNI	mouse	↑	Contribute to the spinal LTP. Mediate the crosstalk of neuron–glia in NP.	Sun et al. (2023)
ATP						
ATP	energy rich phosphate compounds	NMOSD	rat	↑	Induce microglial activation. Mediate the development of mechanical allodynia.	Ishikura et al. (2021)
Enzymes						
MMP-2	protease	SNL	mouse and rat	↑	Maintain NP through IL-1β cleavage and astrocyte activation.	Kawasaki et al. (2008)
Proteins						
Hevin/Sparcl1	glycoproteins	CCI	mouse	↑	Regulate synaptic plasticity and pain via non-NMDARs. Induce central sensitization and mechanical pain.	Chen G. et al. (2022)
SPOCK2	proteoglycan	CCI	rat	↑	Regulate the activation of MMP-2. Affects ERK1/2 activation and IL-1β production. Promote the development of NP.	Wang et al. (2024b)
C3	complement	CCI	rat	↑	Regulate the activation and polarization of microglia. Lead to the development of NP.	Mou et al. (2022)
CAV-1	mosaic protein	SMIR	rat	↑	Upregulate Cx43 expression. Induce nociceptive hypersensitivity.	Hua et al. (2022)
LCN2	lipocalin	CPSP	mouse	↑	Key molecule in CPSP development.	Nakamoto and Tokuyama (2024)
Vascular endothelial growth factor						
VEGF-A	growth factor	CIN	mouse	↑	Activate neuronal firing and induces pain by VEGFR-1 stimulation.	Micheli et al. (2021)

↑, upregulated. IL-, interleukin-; CCI, chronic constriction injury; SNI, spared nerve injury; LTP, long-term potentiation; NP, neuropathic pain; NMOSD, neuromyelitis optica spectrum disorder; MMP-2, matrix metalloproteinase 2; SNL, spinal nerve ligation; Hevin/Sparcl1, high endothelial venule protein/SPARC-like 1; SPOCK2, SPARC (osteonectin), cwcv and kazal-like domains proteoglycan 2; C3, complement 3; CAV-1, caveolin-1; SMIR, skin/muscle incision and retraction; Cx43, connexin 43; LCN2, lipocalin 2; CPSP, central poststroke pain; VEGF-A, vascular endothelial growth factor A; CIN, chemotherapy-induced neuropathy; VEGFR-1, vascular endothelial growth factor receptor-1.

Interleukins (ILs) are a group of cytokines produced by macrophages and lymphocytes that play important roles in cell proliferation, maturation, migration and adhesion; immune cell differentiation and activation; and proinflammatory and anti-inflammatory activities (Brocker et al., 2010). The interleukin-1 (IL-1) family was the first to be identified and has been extensively studied. This family consists of 11 cytokines, and available evidence suggests that one member, IL-1β, is upregulated in a variety of chronic pain conditions. Zhang et al. (2005) found that bone cancer can activate spinal astrocytes and that activated astrocytes can release IL-1β, which promotes the development and maintenance of persistent hyperalgesia. In the CCI-induced NP model, the expression of the inflammasome NLRP3 in spinal astrocytes is increased (Green-Fulgham et al., 2024). In response to NLRP3, pro-IL-1β is cleaved into the active form of IL-1β and released, thus participating in the development of mechanical allodynia (Green-Fulgham et al., 2024). The interleukin-17 (IL-17) family comprises important proinflammatory cytokines. IL-17 was found to be expressed mainly in astrocytes in the superficial spinal dorsal horn, and interleukin 17 Receptor A (IL-17RA) is expressed mainly in neurons and to a lesser extent in astrocytes (Sun et al., 2023). This localization provides a cellular basis for neuron–astrocyte interactions. IL-17 produced by astrocytes may regulate spinal neuroplasticity directly through IL-17RA-expressing neurons or indirectly through IL-17RA-expressing spinal cord glial cells, leading to NP (Figure 1). The application of an anti-IL-17 neutralizing antibody inhibits spinal long term potentiation (LTP) of C-fiber-evoked field potentials and relieves mechanical allodynia (Sun et al., 2023).

Astrocytes also express a variety of proteins. For example, high endothelial venule protein/SPARC-like 1 (hevin/Sparcl1) secreted by reactive astrocytes is able to induce central sensitization and mechanical pain by regulating Glun2B-containing NMDARs in SDH neurons (Chen G. et al., 2022). The expression levels of SPARC (osteonectin), cwcv and kazal-like domains proteoglycan 2 (SPOCK2) in the spinal cord were significantly increased after CCI (Wang et al., 2024b). SPOCK2 can interact with membrane-type 1 matrix metalloproteinase (MT1-MMP14) to regulate the activation of matrix metalloproteinase 2 (MMP-2), thereby affecting ERK1/2 activation and IL-1β production in astrocytes to promote the development of NP (Wang et al., 2024b). CCI leads to increased expression of complement 3 (C3) in astrocytes and its receptor C3aR in microglia in the SDH, which promotes the M1-type polarization of microglia through the formation of crosstalk between astrocytes and microglia via C3/C3aR, leading to the development of NP (Mou et al., 2022) (Figure 1). In the skin/muscle incision and retraction (SMIR) rat model of chronic postsurgical pain, adenosine monophosphate (AMP)-exchange proteins directly activated by cAMP1 (EPAC-1) and caveolin-1 (CAV-1) are overexpressed, particularly in spinal astrocytes and microglia. CAV-1 overexpression upregulates Cx43 expression (Hua et al., 2022). CAV-1 mediates the functional coupling of microglia, astrocytes, and neurons through the cAMP/EPAC-1 pathway, thereby inducing nociceptive hypersensitivity (Hua et al., 2022). In a model of central poststroke pain (CPSP) induced by bilateral common carotid occlusion (BCAO), the expression level of SDH astrocyte-derived lipocalin 2 (LCN2), which is involved in the development of CPSP, is increased (Nakamoto and Tokuyama, 2024).

In the CNS, vascular endothelial growth factor A (VEGF-A) plays a role in regulating the microvessel density and vascular permeability (Lange et al., 2016). Previous studies have suggested distinct roles for VEGF-A in pain signaling, including both algesic and analgesic effects. Micheli et al. (2021) have shown that VEGF-A strongly stimulates the activity of spinal nociceptive neurons through its receptor vascular endothelial growth factor receptor-1 (VEGFR-1). In the SDH of oxaliplatin-induced NP mice, VEGF-A expression is increased in astrocytes, whereas VEGFR-1 is expressed mainly in neurons, suggesting that the astrocyte–neuron interaction mediated by VEGF-A/VEGFR-1 is involved in the pathophysiological process of NP (Figure 1).

#### 2.1.4 Changes in genetic information are involved in the development of NP

Alterations in multiple types of genetic information in astrocytes are also involved in NP. Fang et al. (2022) reported that CCI leads to the downregulation of an SDH astrocyte phosphatase gene (PTEN) and that PTEN inhibition impairs the dephosphorylation of enzymes (e.g., HMGCR) in the cholesterol biosynthesis pathway, which, in turn, leads to cholesterol deficits and neuroglial cell activation. Increasing PTEN expression may accelerate cholesterol biosynthesis and reduce glial cell activation, thereby alleviating NP. MiR-125a-5p is expressed in astrocyte-derived extracellular vesicles and can mediate astrocyte function to regulate neuronal communication. Kasimu et al. (2022) reported that type 2 diabetes mellitus (T2DM) can induce a decrease in the expression of miR-125a-5p in spinal astrocytes. The upregulation of miR-125a-5p can alleviate diabetic peripheral neuropathy (DPN) by inhibiting the activation of astrocytes and tumor necrosis factor receptor associated factor 6 (TRAF6) expression (Kasimu et al., 2022). In addition, T2DM can also induce a decrease in the expression of miR-503-5p in spinal astrocytes, and the overexpression of miR-503-5p can alleviate DPN by reducing Septin 9 (SEPT9) expression (Guo et al., 2024). Zhang et al. (2021) observed that the expression of the lncRNA PVT1 is increased in spinal astrocytes after SCI and is involved in the occurrence of NP. This study confirmed that PVT1 is a competitive endogenous RNA (ceRNA) for miR-186-5p and that inhibition of lncRNA PVT1 expression could inhibit the activation of spinal astrocytes and alleviate NP in SCI rats by upregulating miR-186-5p expression and downregulating CXCL13/CXCR5 expression.

#### 2.1.5 Alterations in cellular function are involved in the development of NP

Astrocytes play important roles in maintaining synaptic function and regulating glutamate and ionic homeostasis. In individuals with NP, these processes are facilitated by astrocyte physiological dysfunction. Li et al. (2021) reported that in a vincristine (VCR)-induced neuropathic pain (VINP) model, VCR promotes the Ca^2+^-dependent phosphorylation of calcium/calmodulin-dependent protein kinase II (CaMKII) and activity of the voltage-dependent calcium channel 3.2 subunit (Ca (V)3.2) and that CaMKII and CaV3.2 may activate astrocytes by increasing the level of intracellular free calcium ([Ca^2+^]i) in the VINP model. These changes promote the Cx43-mediated release of inflammatory factors in spinal astrocytes, leading to the development of NP (Li et al., 2021).

Typically, senescent cells exhibit a unique secretory phenotype that results in the production of a variety of cytokines, chemokines, growth factors, and proteases. Previous studies have shown that senescent astrocytes express IL-1β and IL-6, suggesting that astrocytes in the senescent state develop a proinflammatory phenotype (Campuzano et al., 2009). Du et al. (2023) reported that spinal astrocyte senescence-like response after sciatic nerve injury is associated with NP. Senolytic treatment can effectively eliminate senescent astrocytes and reduce the expression of proinflammatory factors, thus reducing the spinal cord neuroinflammatory response and hypersensitivity after CCI (Du et al., 2023). Additionally, they confirmed that this senescence is related to the expression of the clusterin (CLU) protein in astrocytes (Du et al., 2023).

As a protective mechanism, autophagy can maintain cell homeostasis and promote survival under stress conditions. Previous studies have suggested that the autophagy ability of astrocytes is impaired in individuals with NP and that promoting astrocyte autophagy has an analgesic effect. Hao et al. (2022) reported that SNL induces spinal autophagy flux, but autophagic degradation is impaired, as evidenced by the elevated accumulation of the autophagy-associated proteins E1-like ubiquitin-activating enzyme autophagy-related gene 7 (ATG7), Beclin1, and microtubule associated protein 1 light chain 3-II (LC3-II)/LC3-I and sequestosome 1 (p62/SQSTM1) in the SDH. Translocator protein (TSPO) can activate autophagy, improve autophagy flux, and exert antioxidant and cytoprotective effects by activating the nuclear silent information regulator T1 (SIRT1)/*α* subunit of peroxisome proliferator-activated receptor-*γ* coactivator-1 (PGC-1α) signaling pathway and the downstream expression of nuclear factor erythroid 2-related factor 2 (Nrf2) and heme oxygenase-1 (HO-1) in the SDH cells (Hao et al., 2022). Huang et al. (2024) demonstrated that sphingosine-1-phosphate (S1P)/Sphingosine-1-phosphate receptor 1 (S1PR1) signaling inhibits the Phosphatidylinositol 3-kinase (PI3K)/protein kinase B (Akt) pathway and impairs autophagy flux in SDH astrocytes in CCI-induced NP mice, leading to the polarization of astrocytes toward the A1 phenotype and the release of multiple chemokines.

#### 2.1.6 Cellular phenotypic transformation is involved in the development of NP

**Figure 2:**
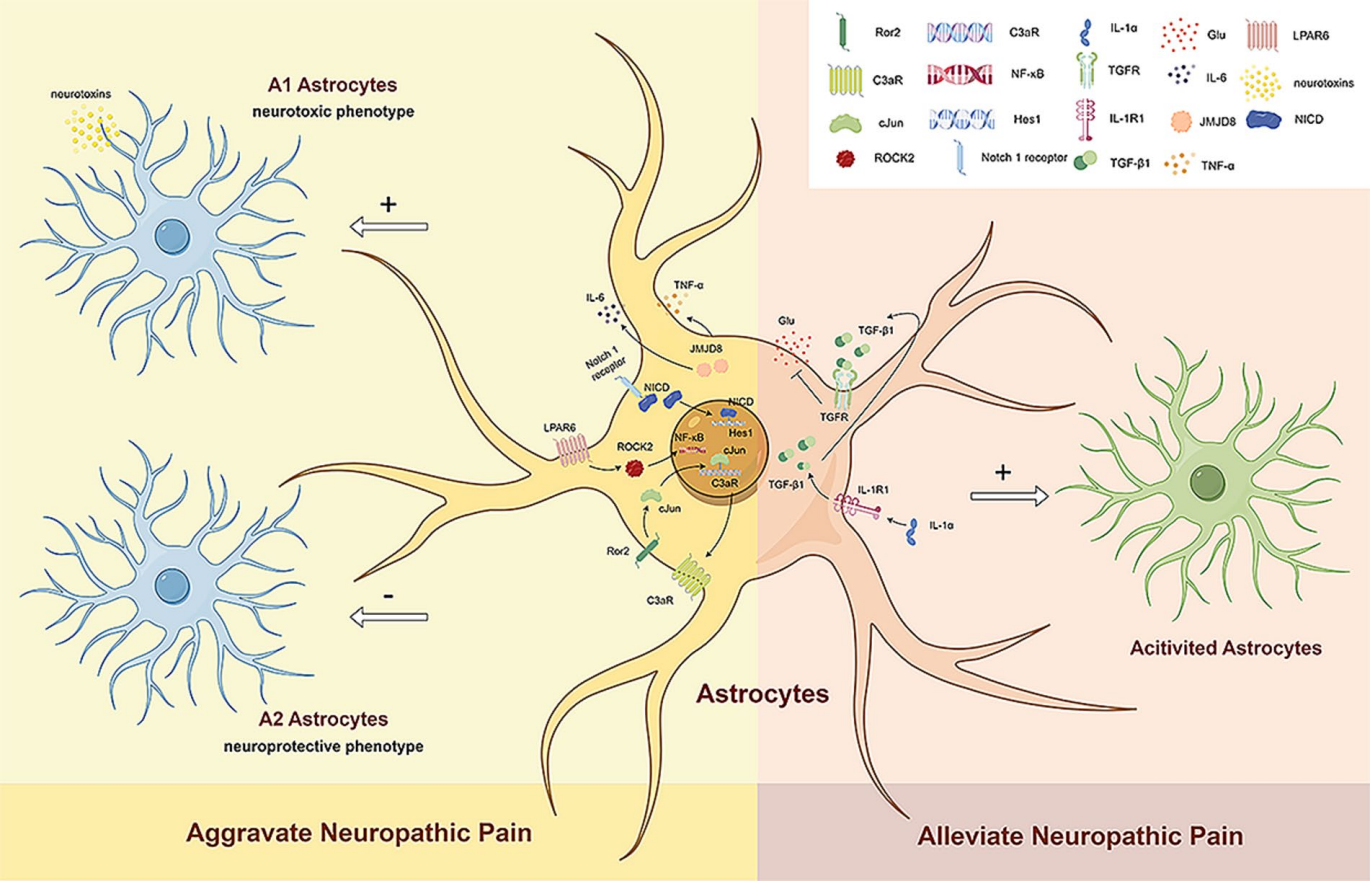
The dual role of reactive astrocytes. Existing studies generally agree that in subjects with NP, activated spinal astrocytes exhibit an A1 phenotype and promote the development of pain. Ror2 in spinal astrocytes regulates c-JUN and C3aR expression, leading to mechanical hyperalgesia and cold allodynia by increasing A1 astrocyte numbers. LPAR6 activation through ROCK2 increases A1 astrocytes and decreases A2 astrocytes via the NF-κB pathway, promoting neuropathic pain. The Notch signaling pathway also induces A1 astrocyte transformation, further advancing neuropathic pain. Reduced JMJD8 expression in spinal dorsal horn astrocytes promotes mechanical allodynia and thermal hyperalgesia by activating A1 astrocytes and increasing TNF-α and IL-6 secretion. However, activated astrocytes can also have beneficial effects on neuropathic pain. Neuronal IL-1α stimulates TGFβ signaling in spinal astrocytes, reducing glutamate release and alleviating NP. Ror2, receptor tyrosine kinase like orphan receptor 2; C3aR, complement C3a receptor; LPAR6, lysophosphatidic acid receptor 6; ROCK2, rho associated coiled-coil containing protein kinase 2; NF-κB, nuclear factor-κB; JMJD8, jumonji domain containing 8; TNF-α, tumor necrosis factor α; IL-6, interleukin-6; NICD, notch intracellular domain; Hes1, hes family BHLH transcription factor 1; IL-1α, interleukin 1α; IL-1R1, interleukin 1 receptor type 1; TGF-β1, transforming growth factor-β1; TGFR, TGFβ receptor; Glu, glumate.

Two different types of reactive astrocytes have been identified in response to neuroinflammation and ischemia induction, namely A1 reactive and A2 reactive astrocytes (Escartin et al., 2021). A1 reactive astrocytes may secrete neurotoxins, leading to the rapid death of neurons and other types of glial cells (Liddelow et al., 2017). In contrast, A2 astrocytes contribute to more efficient tissue healing and neuronal preservation (Liddelow et al., 2017). Although astrocytes may exist in more than a simple binary activation state under pathological conditions (Escartin et al., 2021), such as the possible existence of A3 astrocytes that express the receptors of specialized pro-resolving mediators (SPMs) and act as a pro-resolving phenotype (Ji et al., 2019). However, recent studies are still based on the specific markers C3 and S100A10 of A1 and A2 astrocytes to identify the mechanisms of reactive astrocyte phenotypic transformation and their role in NP. Existing studies generally agree that in subjects with NP, activated spinal astrocytes exhibit an A1 phenotype and promote the development of pain (Figure 2).

Li D. Y. et al. (2022) analyzed a paclitaxel-induced neuropathic pain (PINP) model and found the activated Notch signaling pathway is able to induce the transformation of spinal A1 astrocytes, which contributes to the development of PINP. Liu et al. (2022) studied a model of chronic post-thoracotomy pain (CPTP) and showed that receptor tyrosine kinase like orphan receptor 2 (Ror2) in spinal astrocytes regulates the expression of c-JUN and C3aR to mediate the transformation of A1/A2 reactive astrocytes (Liu et al., 2022). Mechanical hyperalgesia and cold allodynia are induced by increasing the number of A1 astrocytes. Fan et al. (2024) reported that nerve injury can increase the expression of lysophosphatidic acid receptor 6 (LPAR6) in SDH astrocytes and activate the NF-κB pathway through rho associated coiled-coil containing protein kinase 2 (ROCK2), thereby increasing the number of A1 astrocytes and reducing the number of A2 astrocytes to promote the progression of NP. Li et al. (2023) showed that decreased expression of jumonji domain containing 8 (JMJD8) in the SDH after CCI promotes mechanical allodynia and thermal hyperalgesia by inducing the activation of A1 astrocytes and the subsequent secretion of TNF-*α* and IL-6.

### 2.2 Positive role of spinal astrocytes in NP

Following injury or disease, CNS astrocyte activation has a dual role (Li et al., 2019). In the development of pain, astrocyte activation occurs later but lasts longer than microglia activation. A large number of evidences suggest that astrocyte activation plays a role in the maintenance of chronic pain (Gao and Ji, 2010; O'Callaghan and Miller, 2010). However, existing evidence suggests that reactive astrocytes play neuroprotective and reparative roles during the initial stages of nerve injury (Di Cesare Mannelli et al., 2014). As a defense mechanism, at an early stage, reactive astrogliosis can increase neuroprotection and provide nutritional support to neurons (Li et al., 2019). Reactive astrocytes proliferate and migrate to form glial scars, which can reduce neuroinflammation and protect nerve tissue from secondary injury (Koyama, 2014). In addition, reactive astrocyte migration plays a positive role in reconstructing the blood–brain barrier and preventing the spread of lesions to surrounding healthy tissues (Lukacova et al., 2021).

Studies have shown that spinal astrocyte activation has beneficial functions in NP. Di Cesare Mannelli et al. (2022) reported that interleukin-1α (IL-1α) secreted by neurons can stimulate transforming growth factor-*β* (TGFβ) signaling in spinal astrocytes and promote neuroprotection. IL-1α can reduce OXA-induced endogenous glutamate release and increase astrocyte activation, thereby alleviating NP by reducing glutamate release from spinal astrocytes. However, the phenotype of activated astrocytes still needs to be further explored (Figure 2).

## 3 Mechanisms of astrocyte-mediated NP therapy

### 3.1 Drugs

Currently, the treatment of NP is based on drugs, of which the first-line drugs include gabapentinoids, tricyclic antidepressants, etc., and opioids, lidocaine or capsaicin, and even botulinum toxin can be considered second- or third-line treatments (Mitsikostas et al., 2022). However, most patients treated in the clinic cannot achieve effective relief through drugs alone, and drug tolerance and toxic side effects arise because of patients’ long-term dependence on drugs. Therefore, in-depth investigations of the mechanism of drug action and the development of new drugs are also the focus of research.

#### 3.1.1 Commonly used drugs modulate astrocyte activation

Pregabalin is a widely used drug for the treatment of chronic pain, especially diabetic neuropathic pain, postherpetic neuralgia, and cancer pain. It has been reported to relieve pain by inhibiting astrocyte activation, reducing intracellular calcium levels, and inhibiting the production of proinflammatory factors. Song et al. (2023) showed that pregabalin can alleviate CCD-induced NP by inhibiting the P2Y2 receptor/TRPV4/calcium signaling pathway and spinal astrocyte activation. Zhang et al. (2022) reported that in rat models of SNI pregabalin can significantly reduce PKC epsilon (PKCε)/TRPV1 expression in superficial spinal dorsal horn reactive astrocytes, thereby reducing the expression of IL-1β and IL-6 and alleviating SNI-induced NP.

Tricyclic antidepressants represented by amitriptyline are the first-line drugs for NP. As mentioned above, spinal Cx43 overexpression contributes to the induction and maintenance of NP by promoting astrocyte coupling and enhancing chemokine release. Jeanson et al. (2016) found *in vitro* astrocyte culture that amitriptyline was able to reduce the Cx43 mediated diffusion of Lucifer yellow in cultured astrocytes in a concentration dependent manner, indicating a clear inhibitory effect on gap junctions channels (GJCs). This inhibitory effect of amitriptyline was significantly enhanced by the combination of mefloquine, and the combination was more effective in relieving CCI-induced mechanical hyperalgesia than amitriptyline alone in the long-term treatment of CCI rats. Although CCI-induced markers of neuroinflammation and glial activation are unaffected by these treatments in dorsal root ganglia and spinal cord, downstream mechanisms may be targeted, and thus connexin inhibition in astrocytes may represent a promising approach to improve treatment of NP by antidepressants. On the contrary, Sanna et al. (2017) showed that amitriptyline did not reduce mechanical allodynia and thermal hyperalgesia in SNI mice, and further promoted astrocyte activation (such as increased expression of GFAP) and astrocyte JNK activation in NP mice. The results of this study suggest that promoting JNK activation in astrocytes is an counteracting pathway for the analgesic response of amitriptyline, supporting the notion that amitriptyline has limited efficacy in the treatment of NP. Therefore, targeting the JNK pathway in astrocytes may be able to improve the analgesic effect of amitriptyline in the treatment of NP.

Duloxetine (DUL), a serotonin noradrenaline neuronal reuptake inhibitor (SNRI), is used to treat depression, chronic musculoskeletal conditions such as fibromyalgia, and NP. DUL is the only potential treatment for chemotherapy-induced peripheral neuropathy (CIPN) recommended by the American Society of Clinical Oncology. Wang et al. (2022) documented that DUL alleviates paclitaxel (PTX)-induced CIPN by inhibiting the upregulation of TRPV1, which in turn inhibits SDH astrocyte activation and the expression of TNF-*α*. Lobos et al. (2023) reported that DUL combined with rosuvastatin can reverse the PTX-induced overexpression of GFAP in spinal astrocytes and increase the efficacy of DUL. This effect might be partially dependent on the inhibition of astrocyte activation. DUL is also one of the first-line drugs for the treatment of painful diabetic neuropathy (PDN). Tawfik et al. (2018) demonstrated that DUL can be used as a neuroprotective agent for sciatic nerve and spinal cord in the mouse model of PDN. DUL can inhibit the activation of spinal astrocytes and microglia, and up-regulate the expression of sciatic nerve growth factor (NGF), thereby alleviating mechanical allodynia and thermal hyperalgesia in PDN mice.

Morphine (MOR) is a classic opioid that is widely used for the management of severe NP. However, it has serious side effects such as tolerance, dependence and respiratory depression. Previous studies have shown that MOR can activate glial cells, leading to the up-regulation and release of pro-inflammatory cytokines/chemokines, which is an important reason for morphine analgesia tolerance (MAT) (Kadhim et al., 2018). In recent years, much of the research on astrocytes and MOR has focused on addressing the limitation of morphine tolerance. IIK7 is a melatonin type 2 (MT2) receptor agonist with antioxidant properties. Kuthati and Wong (2024) showed that the co-administration of IIK7 and MOR reversed the reduction of Nrf2 and HO-1 expression induced by MOR and inhibited the expression of pro-inflammatory cytokines. At the same time, the co-administration of IIK7 and MOR delayed the development of MAT in partial sciatic nerve transection (PSNT) rats by inhibiting the activation of astrocytes and microglia. A single high-dose injection of IIK7 was also able to effectively reverse preexisting MAT. N-palmitoylethanolamine (PEA) is a natural fatty-acid ethanolamide with anti-inflammatory, neuroprotective and immunomodulatory effects. The co-administration of ultramicronized PEA and MOR can effectively reduce the increase of GFAP expression in astrocytes caused by MOR, and delay the occurrence of morphine tolerance by regulating the activation of astrocytes (Micheli et al., 2024).

Tramadol, a weak opioid and serotonin-norepinephrine reuptake inhibitor, is used as a second-line treatment for NP (Moisset, 2024). Sakakiyama et al. (2014) showed that tramadol can activate *μ*-opioid receptors to exert acute analgesia, and prevent and relieve NP by affecting the activation of spinal astrocytes. Repeated administration of tramadol was able to inhibit partial sciatic nerve ligation (pSNL)-induced activation of spinal astrocytes, but had no significant effect on microglial activation. This inhibitory effect is achieved by increasing the levels of norepinephrine (NA) and activating *α*2-adrenoceptors (α2-ARs) on astrocytes, thereby inhibiting astrocyte activation and reducing the release of inflammatory factors. In addition, repeated tramadol administration did not exhibit the analgesic tolerance and opioid-induced hypersensitivity observed with morphine.

#### 3.1.2 New drugs for different types of NP

A variety of novel drugs attenuate peripheral nerve injury-induced NP by inhibiting the activation of signaling pathways and receptor activation in spinal astrocytes, which in turn inhibits astrocyte activation and downregulates the expression of inflammatory factors, chemokines, angiogenic factors, and glycolytic enzymes (Wen et al., 2021; Cai et al., 2022; Dang et al., 2022; Iamjan et al., 2023; Negah et al., 2023; Pang et al., 2023; Sung et al., 2023; Zhang H. et al., 2023; Zhou et al., 2023; Wen et al., 2024). However, the site of action of the novel drug still needs to be clarified. *β*-Elemene, the main component of the traditional Chinese medicine Curcuma wenyujin, can inhibit ERK activation in SDH astrocytes and the subsequent upregulation of proinflammatory cytokines, thereby alleviating SNI-induced NP (Ma et al., 2022). Elemene also exerts an analgesic effect by downregulating the expression of NMYC downstream-regulated gene 2 (NDRG2) in spinal astrocytes (Ma et al., 2021). Celastrol can inhibit the activation of spinal astrocytes and microglia and the expression of inflammatory factors (TNF-*α*, IL-1β, and IL-6) by inhibiting the TLR4/NF-κB signaling pathway, thus alleviating NP in CCI rats (Jin et al., 2021).

The combined application of minocycline (MC) and botulinum toxin (BoNT) can induce the expression of SIRT1, thereby inactivating the NF-κB, P53 and PI3K/AKT signaling pathways; inhibiting the inflammatory response and oxidative stress of spinal glial cells; and alleviating SCI-induced NP (Yu et al., 2021).

Cancer is a major public health problem, and patients often require chemotherapy. Chemotherapy-induced peripheral neuropathy (CIPN) is a common and serious side effect. The current recommendations for drug treatment of CIPN come from other types of NP or studies with low level of evidence, and the first-line drug is single. Therefore, exploring drugs that can prevent or treat CIPN is important. JI017 is a compound derived from *Aconitum carmichaelii*, Angelica gigas, and *Zingiber officinale*. Lee et al. (2021) reported that JI017 can also inhibit the activation of SDH astrocytes by inhibiting the expression of TRPV1 in the superficial area of the SDH, thereby attenuating OXA-induced cold allodynia. Emodin (Yang et al., 2022) and resveratrol (Dong et al., 2022) alleviate OXA-induced NP by reducing cyclooxygenase-2 (COX2)-mediated oxidative stress and inflammatory responses and inhibiting spinal astrocyte activation.

Cilostazol is an antiplatelet agent with vasodilating properties that has been used in the treatment of peripheral arterial disease, cerebrovascular disease, percutaneous coronary intervention, etc. Existing studies have found that cilostazol may help to relieve diabetic peripheral neuropathy (DPN). Cheng et al. (2022) reported that cilostazol attenuates DPN-induced mechanical allodynia by reversing the downregulation of SDH astrocyte expression, inhibiting the activation of microglia, and reducing the dysregulation of NaV expression in the dorsal root ganglia (DRG).

#### 3.1.3 Novel drugs modulate subtypes of reactive astrocytes

As discussed previously, A1 reactive astrocytes may promote NP by causing the release of molecules such as proinflammatory cytokines, chemokines, and intracellular kinases, while A2 reactive astrocytes may inhibit the progression of NP by secreting neuroprotective factors that promote neuronal survival. Thus, interfering with the A1 phenotypic transformation of astrocytes activation or modulating the transition of reactive astrocytes from pain-promoting to neuroprotective types may become a potential therapeutic strategy for NP.

In recent years, a variety of novel drugs have been found to promote the transformation of deleterious astrocytes (e.g., A1) to beneficial astrocytes (e.g., A2) by targeting or modulating specific pathways. Vitexin is a flavonoid compound with anti-inflammatory and analgesic effects. Huang et al. (2024) showed that vitexin could attenuate the down-regulation of PI3K/Akt signaling induced by S1P/S1PR1 signaling in astrocytes, restore autophagic flux in astrocytes. Thus, it could inhibit the polarization of A1 astrocytes and promote the polarization of A2 astrocytes, and reduce the secretion of chemokines, thereby alleviating NP caused by nerve injury. Daphnetin, an active ingredient extracted from Daphne Korean Nakai, a plant of the genus Daphne, has analgesic and anti-inflammatory activities. Zhang et al. (2023b) revealed that daphnetin inhibits the activation of spinal astrocytes, microglia, and neurons and blocks astrocyte–neuron and neuron–microglia crosstalk by inhibiting the expression of inflammatory factors and chemokines. It also induces the transformation of M1 microglia and A1 astrocytes into M2 microglia and A2 astrocytes, respectively, to improve TNF-α-induced NP. Another study by the same group reported that daphnetin inhibits the upregulation of TLR4 and the phosphorylation of IKBα (inhibitor of NF-κB) in the spinal cord of rats subjected to CCI and reduces the release of NF-κB, which indirectly inhibits CXC chemokine receptor type 2 (CXCR2) activation in SDH neurons and reduces astrocyte activation and CXC chemokine ligand type 1 (CXCL1) release, thus exerting analgesic effects (Zhang et al., 2023a). Rao et al. (2024) reported that the combined application of tetramethylpyrazine (TMPZ) and astragaloside iv (AGS-IV) could reduce the A1 polarization of spinal astrocytes and enhance A2 polarization by affecting the lncRNA OIP5-AS1-Sirt1-NF-κB pathway, thus reducing SCI-induced NP.

It is of great significance to further study the mechanism of A1/A2 astrocyte phenotypic transformation and explore new therapeutic strategies accordingly.

### 3.2 Neuromodulation technology

#### 3.2.1 Electrical nerve stimulation techniques

Various electrical nerve stimulation techniques, such as peripheral nerve stimulation (PNS) and spinal cord stimulation (SCS), have been used for the treatment of NP in patients for whom pharmacological treatments are ineffective or who cannot tolerate pharmacological treatments. The available evidence suggests that spinal astrocytes play an important role in the analgesic mechanism of several electrical nerve stimulation techniques.

Wong et al. (2022) demonstrated that sciatic nerve stimulation (SNS) at 2 Hz and 20 Hz alleviates nerve root ligation (NRL)-induced acute NP by modulating neuroinflammation, facilitating downstream pain inhibition, decreasing the expression of inflammatory proteins, and inhibiting spinal astrocyte proliferation and microglial activation.

Spinal cord stimulation is a safe and effective clinical neuromodulation technique for the treatment of many types of chronic pain, such as complex regional pain syndrome (CRPS), failed back surgery syndrome (FBSS), and peripheral neuropathy (Cameron, 2004). The development of traditional SCS therapy stems from the theory of pain gating, which states that by applying electrical stimulation to the dorsal column of the spinal cord, large-diameter afferent fibers can be activated, thereby inhibiting peripheral pain signals (Melzack and Wall, 1965; Shealy et al., 1967). The existing evidence generally suggests that SCS can inhibit or relieve pain by blocking pain signal transmission, interfering with pain pathways, activating opioid pathways, stimulating the locus coeruleus and regulating *γ*-aminobutyric acid (GABA) (Li S. L. et al., 2022), but its analgesic mechanism is not completely understood.

In recent years, new technologies, such as high-frequency electrical stimulation, closed-loop electrical stimulation, dorsal root ganglion (DRG) electrical stimulation, and different target multiplexing (DTM), have emerged to improve the clinical efficacy of and patient satisfaction with SCS. Several lines of evidence have shown that SCS can target astrocytes to relieve NP. Kilohertz high-frequency electrical stimulation can inhibit the activation of SDH neurons and glia, especially astrocytes and microglia, by suppressing the activation of the TRPV1/NMDAR2B signaling pathway and ultimately alleviating CCI-induced NP (Fang et al., 2024). A DTM programmed-mode SCS approach was developed based on molecular biology research using multiple electrical signals to target microglia, astrocytes and other glial cells and neurons to rebalance their interactions (Cedeño et al., 2020; Vallejo et al., 2020; Tilley et al., 2022). It can also regulate the expression of a variety of proteins and reverse ion transport and signal transduction disorders related to the NP model. This method has been successfully translated into clinical practice, and studies have confirmed that it has a better effect on relieving chronic refractory low back and leg pain than traditional SCS (Fishman et al., 2021).

However, evidence also indicates that spinal astrocytes may be an important component of pain ‘gating’ and that the activation of astrocytes can help to inhibit pain (Xu et al., 2021). Electrical stimulation of peripheral Aβ fibers in naïve mice activates spinal astrocytes, which release various neurotransmitters that bind to astrocytes, such as glutamate and ATP, thereby inducing long-term depression (LTD) in neurokinin 1 receptor-positive (NK1R) projection neurons and suppressing pain through the activation of endogenous adenosine energy mechanisms (Xu et al., 2021).

#### 3.2.2 Photobiomodulation

Photobiomodulation (PBM) is a treatment method that uses very-low-energy light for stimulation. In recent years, many studies have emphasized the therapeutic role of PBM in NP. Zhang Z. et al. (2023) reported that PBM inhibits the activation of the NF-κB pathway, reduces downstream CXCL10 expression, and significantly inhibits the activation of spinal astrocytes and microglia, thereby alleviating NP after SCI.

### 3.3 Exercise

Exercise is one of the important treatment options for patients with NP, which has a positive effect on preventing chronic diseases and promoting physical and mental health (Leitzelar and Koltyn, 2021). Exercise can relieve NP by changing the activity of microglia, preventing the up-regulation of pro-inflammatory cytokines and promoting the release of anti-inflammatory cytokines, affecting the expression of neurotrophic factors to affect nerve regeneration, inhibiting the dopaminergic system, and increasing endogenous opioids (Leitzelar and Koltyn, 2021; Zheng et al., 2024; Zhu et al., 2024). Current evidence suggests that inhibition of astrocyte activation and reversal of abnormal expression of GFAP are involved in the analgesic mechanism of exercise. Almeida et al. (2015) demonstrated that swimming for 5 weeks could reverse the activation of spinal astrocytes and microglia, thereby reversing mechanical allodynia. TRAF6, which is expressed on astrocytes, is involved in astrocyte activation and maintains NP by integrating TNF-*α* and IL-1β signaling and activating the JNK/CCL2 pathway. Sumizono et al. (2022) found that continuous treadmill exercise for 5 weeks can inhibit the expression of TRAF6 in spinal astrocytes, thereby inhibiting the activation of astrocytes and relieving NP. Another study by the authors also found an association between exercise frequency and glial cell activation, BDNF and endogenous opioid expression (Sumizono et al., 2018). Both low-frequency exercise (3 days a week for 5 weeks) and high-frequency exercise (5 days a week for 5 weeks) can inhibit the activation of spinal astrocytes and microglia by affecting the expression of BDNF, and relieve NP. However, high-frequency but not low-frequency exercise decreased BDNF expression and increased endogenous opioids, suggesting that some intracellular expressions associated with NP may be modulated by exercise frequency. Wang et al. (2024a) demonstrated that treadmill exercise could reverse SNI-induced NP by inhibiting the expression and function of complement component 3 (C3) in spinal astrocytes. Cheng et al. (2023) reported that exercise combined with the administration of adipose-derived stem cells (ADSCs) not only facilitates the early recovery of motor function but also partially alleviates SCI-induced NP by reducing the number of SDH astrocytes. At present, the relationship between spinal astrocytes and exercise still needs to be further explored.

## 4 Conclusion

A growing body of research has provided a rationale for the importance of spinal astrocytes in NP and has contributed to the understanding of the complex mechanisms underlying the formation and maintenance of NP. Reactive astrocytes have a dual role. As an early defense mechanism, astrocyte activation can enhance neuroprotection and stimulate neurogenesis. Thus, complete inhibition of astrocyte activation may not be positive. However, long-term and chronic astrocyte activation participates in the crosstalk with other glial cells and neurons through morphological and functional alterations, thereby aggravating the development of pain and the maintenance of NP. An in-depth study of the functions and mechanisms of spinal astrocytes in NP is expected to provide new ideas and methods for pain treatment.

In clinical practice, the treatment of NP requires a multimodal and multidirectional approach. Astrocytes play a very important role in NP treatment. Continued in-depth studies of astrocyte-mediated NP therapeutic targets will provide new avenues for the development of more effective analgesics.

## Glossary

C3aR: complement C3a receptor
CCI: chronic constriction injury
CNS: central nervous system
CXCL-: C-X-C motif chemokine ligand-
CXCR-: C-X-C motif chemokine receptor-
ERK: extracellular regulated protein kinase
GFAP: glial fibrillary acidic protein
IL-: interleukin-
JAK: Janus kinase
MAPK: mitogen-activated protein kinase
mTOR: mammalian target of rapamycin
NF-κB: nuclear factor kappa-light-chain-enhancer of activated B cells
NP: neuropathic pain
OXA: oxaliplatin
SCI: spinal cord injury
SDH: spinal dorsal horn
SNI: spared nerve injury
SNL: spinal nerve ligation
STAT3: signal transducer and activator of transcription 3
TNFα: tumor necrosis factor-α
TRPM7: transient receptor potential melastatin 7
